# Persistent glutamate increase in TANGO2 deficiency with normalization after vitamin supplementation: first documentation by MR spectroscopy

**DOI:** 10.1186/s13023-026-04338-x

**Published:** 2026-03-30

**Authors:** Taku Omata, Ryo Sugiyama, Yuko Shimizu-Motohashi, Jun-ichi Takanashi

**Affiliations:** 1 Department of Pediatrics, Tokyo Women’s Medical University Yachiyo Medical Center, Yachiyo, Japan; 2 Department of Child Neurology, National Center Hospital, National Center of Neurology and Psychiatry, Kodaira, Tokyo Japan

**Keywords:** TANGO2 deficiency, Magnetic resonance spectroscopy, Glutamate, Glu–gln cycling

## Abstract

**Background:**

TANGO2 deficiency is a rare inherited metabolic disorder characterized by recurrent metabolic crises, rhabdomyolysis, arrhythmias, and encephalopathy. Recent studies suggest that TANGO2 functions as a mitochondrial lipid-acyl-CoA handling protein. Nonetheless, the cerebral metabolic abnormalities underlying neurological deterioration remain elusive. In this study, we present the first longitudinal evaluation of brain metabolism using proton magnetic resonance spectroscopy (MRS) before and after vitamin therapy, during the acute, subacute, and post-treatment phases, highlighting improved clinical manifestations following treatment.

**Methods:**

We longitudinally evaluated cerebral metabolites using MRS during the acute, subacute, and post-treatment phases.

**Results:**

A 2-year-old boy with genetically confirmed TANGO2 deficiency presented with a metabolic crisis, status epilepticus, and rhabdomyolysis (CK peak, 282,695 U/L). Despite the absence of structural abnormalities on the magnetic resonance imaging conducted on day 2, MRS revealed markedly increased glutamate (Glu; 11.51 mM; reference: 6.70 ± 0.59) with normal glutamine (Gln). On day 50, despite clinical stabilization, Glu levels remained abnormally high (9.29 mM), indicating a persistent dysfunction of the cerebral Glu–Gln cycle. Upon initiation of vitamin supplementation, including that of all eight B vitamins (i.e. B₁, B₂, B₃, B₅, B₆, B₇, B₉, and B₁₂) at the age of 3, no further crises occurred. Follow-up MRS at the age of 4 demonstrated Glu normalization (7.20 mM; reference: 6.50 ± 0.70).

**Conclusions:**

This case provides pioneering in vivo evidence of sustained cerebral Glu accumulation in a patient with TANGO2 deficiency along with improvement after metabolic therapy. Our results suggest that the impairment of energy-dependent Glu–Gln cycling and astrocytic Glu clearance might constitute a potential mechanism underlying neurological vulnerability in TANGO2 deficiency. Longitudinal MRS may allow metabolic instability and treatment responsiveness monitoring in this disorder.

## Background

TANGO2 deficiency is a rare hereditary metabolic disorder characterized by recurrent metabolic crises, rhabdomyolysis, potentially fatal arrhythmias, and episodes of encephalopathy [1, 2, 3, 4]. Despite the burgeoning acknowledgement of the clinical phenotype, the fundamental biochemical mechanisms remain elusive. Recent mechanistic investigations have revealed that TANGO2 localizes to the mitochondria and operates as a lipid-derived acyl-CoA binding protein, implicating impaired mitochondrial energy metabolism as a pivotal contributor to metabolic decompensation [5, 6, 7].

Neurologically, patients might experience seizures, acute encephalopathy, movement disorders, or long-term developmental impairments. Nevertheless, in vivo data on cerebral metabolic dysfunction are exceedingly scarce, and the neurochemical basis underlying acute deterioration remains ambiguous. Proton magnetic resonance spectroscopy (MRS) facilitates the quantification of metabolites, such as glutamate (Glu) and glutamine (Gln), which reflect Glu (in neurons)–Gln (in astrocytes) cycling [8].

In this study, we present the inaugural case of TANGO2 deficiency, in which longitudinal MRS during the acute, subacute, and post-treatment phases exhibited a sustained cerebral Glu level increase with subsequent normalization upon vitamin-based metabolic therapy. Our results provide novel mechanistic insights into the neurometabolic vulnerability of TANGO2 deficiency.

## Methods

### Clinical assessment

Clinical and biochemical data were procured in close temporal proximity to the MRS assessments during the acute crisis (approximately day 2), subacute phase (approximately day 50), and follow-up after starting vitamin-based metabolic therapy at the age of 4 years. Vitamin supplementation consisted of a multivitamin preparation (PANVITAN POWDER for Prescription®) and vitamin B7 (biotin). PANVITAN contains vitamins A, B1, B2, B3, B5, B6, B9, B12, C, D2, and E. The patient received PANVITAN powder 0.3 g/day and biotin 0.1 mg/day. PANVITAN was selected because a preparation containing only B-complex vitamins was not available (Table 1).

**Table 1:** Longitudinal changes in cerebral metabolites

Metabolite (mM)	Acute phase, 2 years (Day 2)	Subacute phase (Day 50)	Follow-up,4 years	Reference range
Glutamate (Glu)	**11.51**	**9.29**	7.20	6.70 ± 0.59 (Day 2–50) 6.50 ± 0.70 (4y)
Glutamine (Gln)	2.54	2.03	1.95	2.10 ± 0.28 (Day 2–50) 1.90 ± 0.30 (4y)
N-acetylaspartate (NAA)	**5.21**	**5.76**	7.21	6.81 ± 0.49 (Day 2–50) 7.80 ± 0.60 (4y)

Abnormal data are indicated in bold.

### Genetic analysis

Whole-exome sequencing identified biallelic pathogenic variants in *TANGO2*, consisting of a frameshift variant (c0.242del, p.Leu81Argfs*16) inherited from the mother and a microdeletion in the paternal allele, as previously reported [9].

### MRS

MRS was performed using a 3.0-T Philips Ingenia CX scanner as part of routine MRI evaluation. A single-voxel short-echo PRESS sequence was acquired from the central semiovale (voxel 4.5 cm^3^). Metabolites including Glu, Gln, and N-acetylaspartate (NAA)creatine (Cr), choline (Cho), were quantified using the LCModel.

### Reference values

Age-matched pediatric MRS data were acquired at our institution using an identical protocol that served as a reference.

## Results

### Acute crisis

In this report, the acute phase refers to the early stage of the metabolic crisis characterized by status epilepticus and impaired consciousness. The subacute phase refers to the recovery phase after clinical stabilization. MRS examinations were performed on day 2 (acute phase) and day 50 (subacute phase) after the onset of the metabolic crisis. The overall clinical timeline of the patient is summarized in Table 2.

**Table 2:** Clinical timeline of the patient

Age/time point	Clinical event
1 year 6 months	Mild developmental delay; episodic ataxia triggered by mild infections
2 years (Day 0)	Metabolic crisis with status epilepticus and rhabdomyolysis
Day 2	Acute-phase MRS: Glu elevation, NAA decrease
Day 50	Subacute-phase MRS: persistent Glu elevation
3 years	Genetic diagnosis of TANGO2 deficiency; vitamin supplementation initiated (PANVITAN + biotin)
4 years	Follow-up MRS showing normalization of Glu; no further metabolic crises after initiation of vitamin supplementation

**Fig. 1:**
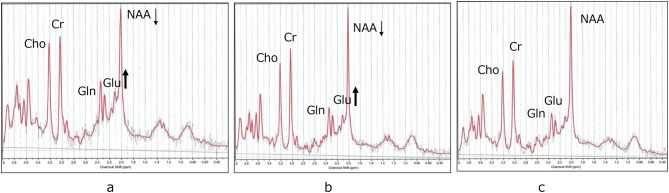
Longitudinal proton MRS of the central semiovale in a patient with TANGO2 deficiency. (**a**) Acute phase (day 2). Short-echo single-voxel PRESS MRS demonstrates markedly increased glutamate levels (Glu 11.51 mM; reference 6.70 ± 0.59 mM). (**b**) Subacute phase (day 50). Despite clinical improvement, Glu remained abnormally high (9.29 mM), indicating the persistent impairment of cerebral Glu handling. (**c**) Follow-up at the age of 4 years. Upon the initiation of vitamin-based metabolic therapy, Glu levels normalized (7.20 mM; reference 6.50 ± 0.70), suggesting metabolic restoration

Before the metabolic crisis, the patient had mild developmental delay and episodic ataxia triggered by mild infections. His expressive vocabulary consisted of approximately 20 meaningful words. At the age of 2 years, the patient presented with status epilepticus, impaired consciousness, and massive rhabdomyolysis (CK peak: 282,695 U/L), consistent with a metabolic crisis. His MRI indicated no structural abnormalities, including diffusion-weighted images. MRS on day 2 demonstrated substantially increased Glu (11.51 mM) relative to the institutional pediatric controls (i.e., 6.70 ± 0.59 mM). Moreover, NAA, a marker for neuronal function, decreased to 5.21 mM (reference: 6.81 ± 0.49 mM) (Fig. 1a, Table 3).

**Table 3:** Vitamin supplementation regimen

Supplement	Formulation	Dose
PANVITAN POWDER for Prescription®	Multivitamin preparation containing vitamins A, B1, B2, B3, B5, B6, B9, B12, C, D2, and E	0.3 g/day
Biotin	Vitamin B7	0.1 mg/day

### Subacute phase

Notwithstanding clinical improvement, MRS on day 50 continued to indicate abnormally increased Glu levels (9.29 mM), indicating persistent dysfunction of the cerebral Glu–Gln cycle. NAA partially recovered, but remained below the reference range (5.76 mM; reference: 6.81 ± 0.49 mM) (Fig. 1b, Table 1). Simultaneously, rhabdomyolysis resolved in this phase (CK 277 U/L).

### Post-treatment phase

After genetic confirmation, vitamin supplementation with a multivitamin preparation (PANVITAN POWDER for Prescription®) and biotin was initiated at the age of 3 years. Subsequently, no further metabolic crises were observed during the follow-up period. At 4 years of age, language development also progressed and the patient was able to produce two-word phrases, although mild developmental delay remained. Follow-up MRS at the age of 4 years demonstrated Glu normalization (7.20 mM; reference: 6.50 ± 0.70), indicating metabolic restoration. Moreover, NAA returned to the normal range (7.21 mM; reference: 7.80 ± 0.60 mM) (Fig. 1c, Table 1).

During the course of the study, no abnormalities were observed in other major metabolites including Gln, Cr, and Cho.

Although a previous publication pertaining to this patient described the related clinical manifestations and muscle MRI results (9), it did not comprise the brain MRS data or the corresponding longitudinal changes. Such neurometabolic findings are presented here for the first time. This study was approved by the Tokyo Women’s Medical University Institutional Review Board (approval number: 3535 R, 2023–0068). Written informed consent was obtained from the mother of the patient.

## Discussion

This case represents the first documentation of sustained cerebral Glu elevation in TANGO2 deficiency and its subsequent improvement after vitamin B complex-based metabolic therapy, demonstrated using serial MRS. The results of this study provide important insights into the neurometabolic phenotype of TANGO2 deficiency.

TANGO2 deficiency is a rare inherited metabolic disease characterized by recurrent metabolic crises, rhabdomyolysis, encephalopathy, and life-threatening arrhythmias. These manifestations are increasingly considered central to its pathophysiology. In the present case, Glu levels were markedly increased in the acute phase (i.e., 11.51 mM on day 2) and remained abnormally high even after clinical stabilization (i.e., 9.29 mM on day 50). The sustained Glu increase observed in this patient differed from the transient increase classically observed in acute encephalopathy with biphasic seizures and late reduced diffusion (AESD) [8, 10]. In AESD, MRS reveals increased Glu only in the acute phase within a few days, followed by increased Gln for approximately 10 days. Although this single case does not allow for mechanistic conclusions, the pattern is consistent with the possibility that energy-dependent processes, including the Glu–Gln cycle and astrocytic Glu uptake, could be vulnerable in TANGO2 deficiency. NAA mildly decreased during the acute and subacute phases but normalized by the follow-up, a pattern potentially consistent with reversible neuronal metabolic dysfunction rather than permanent neuronal loss, particularly given the absence of structural abnormalities on MRI.

Importantly, Glu levels normalized after the initiation of vitamin supplementation, including all B vitamins. Although vitamin therapy reportedly improves clinical outcomes in TANGO2 deficiency [3], this study demonstrates first a temporal association between vitamin supplementation and improved cerebral metabolic abnormalities. B-complex vitamins, serving as key cofactors in multiple mitochondrial and amino acid metabolic pathways, might improve energy-dependent Glu handling in neurons and astrocytes. Further studies are warranted to clarify whether Glu dynamics may serve as a potential biomarker for metabolic stabilization or treatment responsiveness in patients with TANGO2 deficiency. Validation in larger cohorts and correlation with peripheral markers may help to confirm its clinical utility. In summary, this case positions persistent cerebral Glu increase as a potential neurometabolic feature of TANGO2 deficiency and demonstrates that vitamin-based metabolic therapy may improve this abnormality. Further documented cases and the incorporation of MRS into metabolic TANGO2 deficiency evaluation might deepen our understanding of the related neuropathology and guide future therapeutic strategies.

Concerning the limitations of this study, it pertains to an individual case report with findings that cannot be extrapolated to all patients with TANGO2 deficiency. Although Glu levels normalized on follow-up MRS performed approximately 1 year after the initiation of vitamin-based metabolic therapy, this temporal correlation did not establish causation. Spontaneous recovery over time or age-related maturation may also have contributed to the observed normalization. More extensive investigation would be required to ascertain whether increased Glu levels represent a consistent neurometabolic feature of TANGO2 deficiency and Glu dynamics may serve as a potential biomarker for treatment efficacy.

## Conclusions

Longitudinal MRS demonstrated persistent cerebral Glu increase with subsequent normalization upon vitamin B complex-based metabolic therapy in a patient with TANGO2 deficiency, indicating that MRS might be useful for monitoring neurometabolic changes in this disorder.

## Data Availability

The datasets generated and analyzed during the current study are available from the corresponding author on reasonable request.

## Abbreviations

AESD: Acute encephalopathy with biphasic seizures and late reduced diffusion
Cho: Choline
CK: Creatine kinase
Cr: Creatine
Gln: Glutamine
Glu: Glutamate
MRS: Magnetic resonance spectroscopy
MRI: Magnetic resonance imaging
NAA: N-acetylaspartate
